# MetaPepticon: automated prediction of anticancer peptides from microbial genomes and metagenomes

**DOI:** 10.7717/peerj.20990

**Published:** 2026-03-27

**Authors:** Ahmet Arıhan Erözden, Nalan Tavşanlı, Gamze Demirel, Nazmiye Ozlem Sanli, Mahmut Çalışkan, Muzaffer Arıkan

**Affiliations:** 1 Biotechnology Division, Department of Biology, Faculty of Science, Istanbul University, Istanbul, Turkey; 2 Institute of Graduate Studies in Science, Istanbul University, Istanbul, Turkey; 3 Department of Medical Biology and Genetics, Faculty of Medicine, Maltepe University, Istanbul, Turkey

**Keywords:** Anticancer peptide, Genomics, Metagenomics, Microbiome, Bioinformatics

## Abstract

**Background:**

Anticancer peptides (ACPs) are increasingly recognized as promising therapeutic candidates due to their ability to selectively target cancer cells. However, the systematic discovery of novel ACPs, particularly from high-throughput sequencing datasets, remains hindered by technical and methodological limitations. Current prediction frameworks require pre-extracted peptide sequences, involve manual preprocessing, and yield variable results, which restricts their applicability for large-scale, data-driven discovery.

**Methods:**

To address these limitations, we developed MetaPepticon, a modular, end-to-end pipeline for the discovery of ACP candidates from diverse sequencing inputs, including raw genomic, metagenomic, transcriptomic, and metatranscriptomic reads, as well as assembled contigs and peptide sequences. MetaPepticon automates quality control, filtering, assembly, small open reading frame prediction, ACP classification using multiple predictive algorithms, and *in silico* toxicity filtering.

**Results:**

MetaPepticon enables scalable and reproducible ACP prediction from raw sequences through integration of multiple predictors within a configurable agreement framework. Applied to 41,171 microbial genomes and 4,072,884 peptides, MetaPepticon identified 10,725 moderate-agreement ACP candidates, including 4,590 novel, non-toxic peptides. MetaPepticon expands the practical applicability of existing ACP prediction methods to high-throughput sequencing data and is freely available at: https://github.com/arikanlab/MetaPepticon.

## Introduction

Cancer remains the second leading cause of death worldwide, following cardiovascular diseases, and is responsible for one in every eight deaths (Siegel, Giaquinto & Jemal, 2024). By 2070, breast and colorectal cancer cases are expected to reach 9.1 million, representing a 131% increase from 2018, driven by demographic changes and rising incidence rates (Soerjomataram & Bray, 2021). The limitations and adverse effects associated with conventional cancer treatments have driven the search for novel therapeutic alternatives (Kaur, Bhardwaj & Gupta, 2023).

Anticancer peptides (ACPs) are short peptide sequences, typically ranging from 10 to 50 amino acids, that selectively target tumor cells and induce cell death through diverse mechanisms (Xie, Liu & Yang, 2020; Nhàn, Yamada & Yamada, 2023). Compared to traditional chemotherapy drugs, ACPs offer significant advantages, including enhanced membrane permeability, a broader range of molecular targets, and reduced adverse effects (Ghaly et al., 2023). These features make ACPs promising alternatives for cancer treatment, increasing interest in their discovery and validation for therapeutic development. Consequently, computational tools for *in silico* prediction and characterization of ACPs have been increasingly developed over the past decade, facilitating initial screening and discovery of novel therapeutic peptides (Akbar et al., 2017; Akbar et al., 2020; Akbar et al., 2022; Agrawal et al., 2021; Liang et al., 2021; Han et al., 2022; Ghaly et al., 2023; Lee & Shin, 2024); Shahid et al., 2025a; Shahid et al., 2025b).

Microbial communities are rich sources of functional peptides, including ACPs (Arıkan, 2023; Abdou et al., 2023; Ma et al., 2023), and advances in sequencing technologies have enabled large-scale exploration of their diversity, producing vast meta-omics datasets (Arıkan & Muth, 2023). While these data provide new opportunities for ACP discovery, several challenges persist, including the lack of automated tools for direct ACP prediction from raw meta-omics data, difficulties in identifying small open reading frames (smORFs) (Durrant & Bhatt, 2021), and inconsistencies among existing prediction algorithms (Arıkan, 2023). Additionally, current ACP prediction tools require peptide sequences as input (Liang et al., 2021; Ghaly et al., 2023), creating a computational bottleneck for microbial genome and metagenome samples. Together, these constraints hinder efficient and reliable ACP prediction, necessitating improved approaches.

To address these challenges, we developed MetaPepticon, a modular, end-to-end computational pipeline for large-scale ACP prediction from diverse inputs, ranging from raw metagenomic sequencing reads to assembled contigs and peptide sequences. MetaPepticon uniquely bridges small open reading frame (smORF) discovery with peptide-level functional prediction, enabling systematic transition from microbial genomic data to prioritize ACP candidates within a single automated workflow. By integrating multiple independent ACP prediction tools, the pipeline implements a configurable, agreement-based prioritization strategy that improves the robustness and interpretability of candidate identification while allowing users to balance sensitivity and specificity. The resulting output is a table of ACP candidates for downstream analyses.

## Materials & Methods

### Pipeline overview

**Figure 1:**
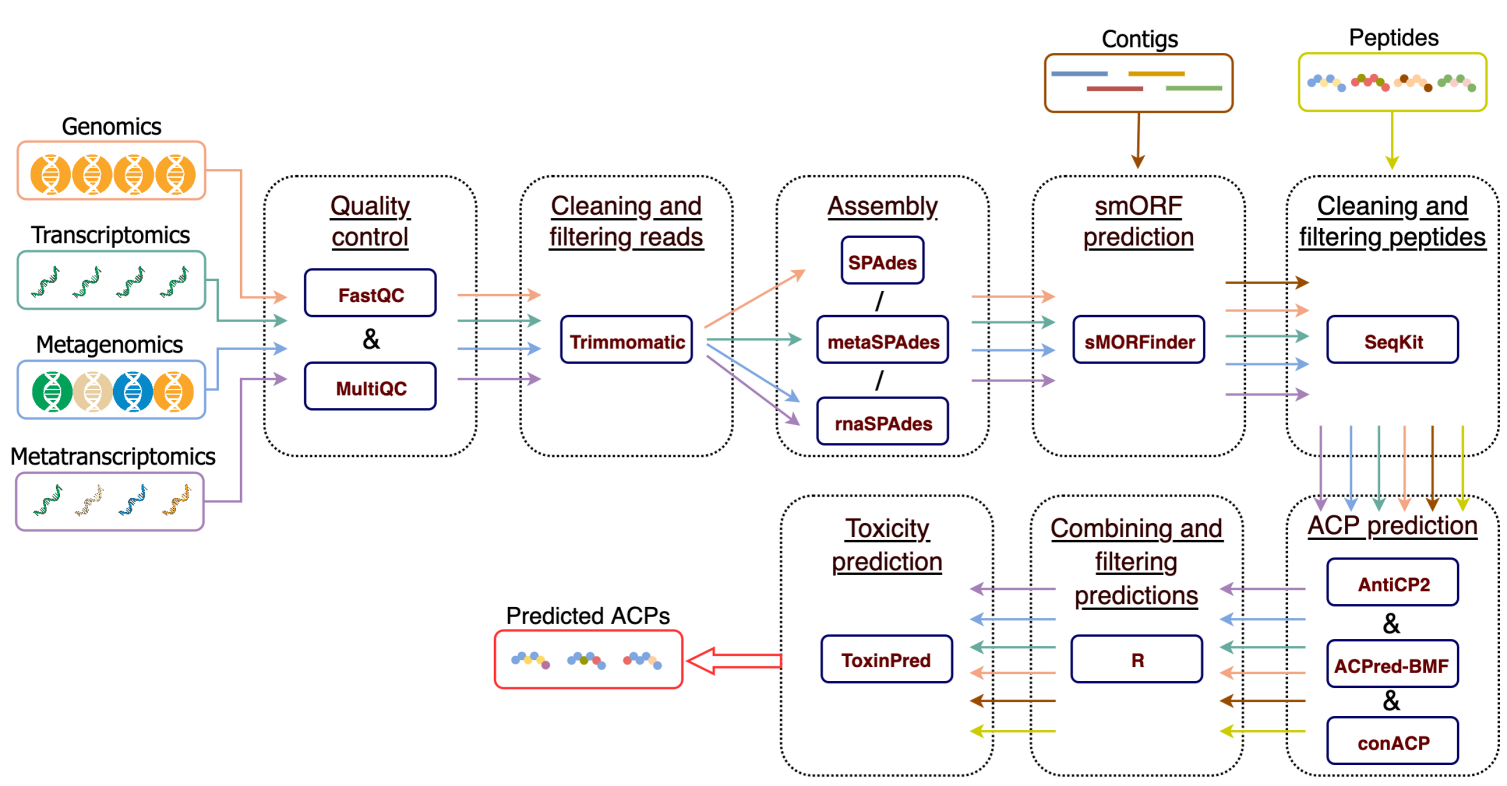
Overview of the MetaPepticon. MetaPepticon takes SG, MG, ST, MT, CO, and PE data as input and produces ACP and toxicity prediction results. The workflow consists of six modules, each optimized for specific input types. Key steps include: quality control, cleaning, and filtering (SG, MG, ST, MT); *de novo* assembly (SG, MG, ST, MT); smORF prediction (SG, MG, ST, MT, CO); peptide cleaning and filtering (SG, MG, ST, MT, CO, PE); ACP prediction (SG, MG, ST, MT, CO, PE); integration and filtering of ACP predictions (SG, MG, ST, MT, CO, PE); and toxicity prediction (SG, MG, ST, MT, CO, PE). The final output file contains peptide sequences alongside prediction results from each tool, customized by user-defined parameters.

MetaPepticon is a modular and reproducible workflow for the discovery of anticancer peptide (ACP) candidates from six input types: (i) single-organism shotgun sequencing (SG), (ii) shotgun metagenomics (MG), (iii) single-organism transcriptomics (ST), (iv) metatranscriptomics (MT), (v) assembled contigs (CO), and (vi) peptide sequences (PE). Implemented in Snakemake (Koster & Rahmann, 2012), each module runs within an isolated conda environment to ensure reproducible dependency management. Depending on the input type, the pipeline selectively activates modules for preprocessing, assembly, smORF prediction, ACP classification, toxicity filtering, and reporting (Fig. 1).

### Input handling and configuration

Users initiate an analysis by placing input files into predefined folders (SG, MG, ST, MT, CO, PE) within MetaPepticon. Then, command line interface or graphical user interface can be used to generate a configuration file. Input file types and relevant parameters are automatically detected and presented to user. The configuration file exposes adjustable parameters including quality thresholds, assembly options, peptide length boundaries and consensus rules for ACP classification.

### Preprocessing and assembly

For raw sequencing reads, quality control is performed with FastQC (Schmieder & Edwards, 2011), followed by adapter and quality trimming using Trimmomatic (Bolger, Lohse & Usadel, 2014) (default: minimum Phred score = 25, minimum read length = 25 bp). Surviving reads are assembled with SPAdes (Bankevich et al., 2012 (for SG), metaSPAdes (for MG), or rnaSPAdes (Bushmanova et al., 2019) (for ST and MT). A minimum contig length (default: 1,000 bp) is applied to exclude short or low-confidence assemblies.

### smORF prediction and peptide filtering

smORFs are predicted using smORFinder (Durrant & Bhatt, 2021), and the resulting peptides are filtered by length (default: 10–50 amino acids) with user-adjustable thresholds. Identical sequences are collapsed to a single representative to remove redundancy and then N-terminal methionine cleavage is performed.

### ACP prediction and agreement strategy

Candidate peptides are evaluated using three independent classifiers: AntiCP2.0 (Agrawal et al., 2021), ACPred-BMF (Han et al., 2022), and ConACP (Lee & Shin, 2024). MetaPepticon then applies a configurable agreement strategy, allowing users to retain ACPs predicted by at least one, two, or all three algorithms, depending on the desired stringency level. Each predictor is used with its original decision rules and default thresholds and MetaPepticon does not modify or recalibrate individual model outputs. Specifically, AntiCP2.0 produces a continuous score between 0 and 1, which is converted to a binary ACP/non-ACP label using the author-recommended threshold (score > 0.5). ACPred-BMF and ConACP provide binary predictions according to their respective default criteria.

Agreement levels are defined by the number of predictors assigning a positive ACP label to a given peptide: low agreement (≥1 predictor), moderate agreement (≥2 predictors), and complete agreement (3/3 predictors). Based on benchmarking across three negative peptide models, moderate agreement may be suitable for general applications, whereas complete agreement may be preferred when minimizing false positives.

### Toxicity filtering

All predicted ACP candidates are screened for potential toxicity with ToxinPred3 (Rathore et al., 2024). Sequences exceeding a configurable toxicity score (ToxinPred3 default: 0.38) are removed from the final dataset.

### Output and reporting

The pipeline generates a final table of predicted ACP candidates, including classifications from each prediction tool and toxicity assessments, as well as intermediate files, detailed logs, and quality control reports.

## Results

### Performance and characteristics of MetaPepticon

We evaluated the performance of MetaPepticon using experimentally validated anticancer peptides (ACPs) from the CancerPPD2 database (Chauhan et al., 2025), together with three complementary negative peptide sets designed to probe predictor behavior under increasing benchmark stringency. All negative sets were length-matched to ACPs (10-50 amino acids) and consisted exclusively of natural amino acids. After filtering for peptide length, amino acid composition, and redundancy, 1,028 ACPs remained from CancerPPD2, and an equal number of peptides were generated for each negative set. Candidate peptides were stratified by predictor agreement level: low-agreement (≥1 predictor), moderate-agreement (≥2 predictors), and complete-agreement (3/3 predictors).

The first negative set comprised composition-matched randomized peptides generated by sampling amino acids according to the empirical frequency distribution of ACPs, producing sequences that preserve global composition but not per-sequence amino acid counts or local patterns. The second set consisted of shuffled peptides, in which each ACP sequence was permuted to preserve exact amino acid composition while disrupting sequence order, representing a more stringent, composition-controlled benchmark. The third negative set comprised random peptides generated using global UniProtKB/Swiss-Prot (Bateman et al., 2023) amino acid frequencies, providing a biologically realistic but compositionally distinct background.

**Figure 2:**
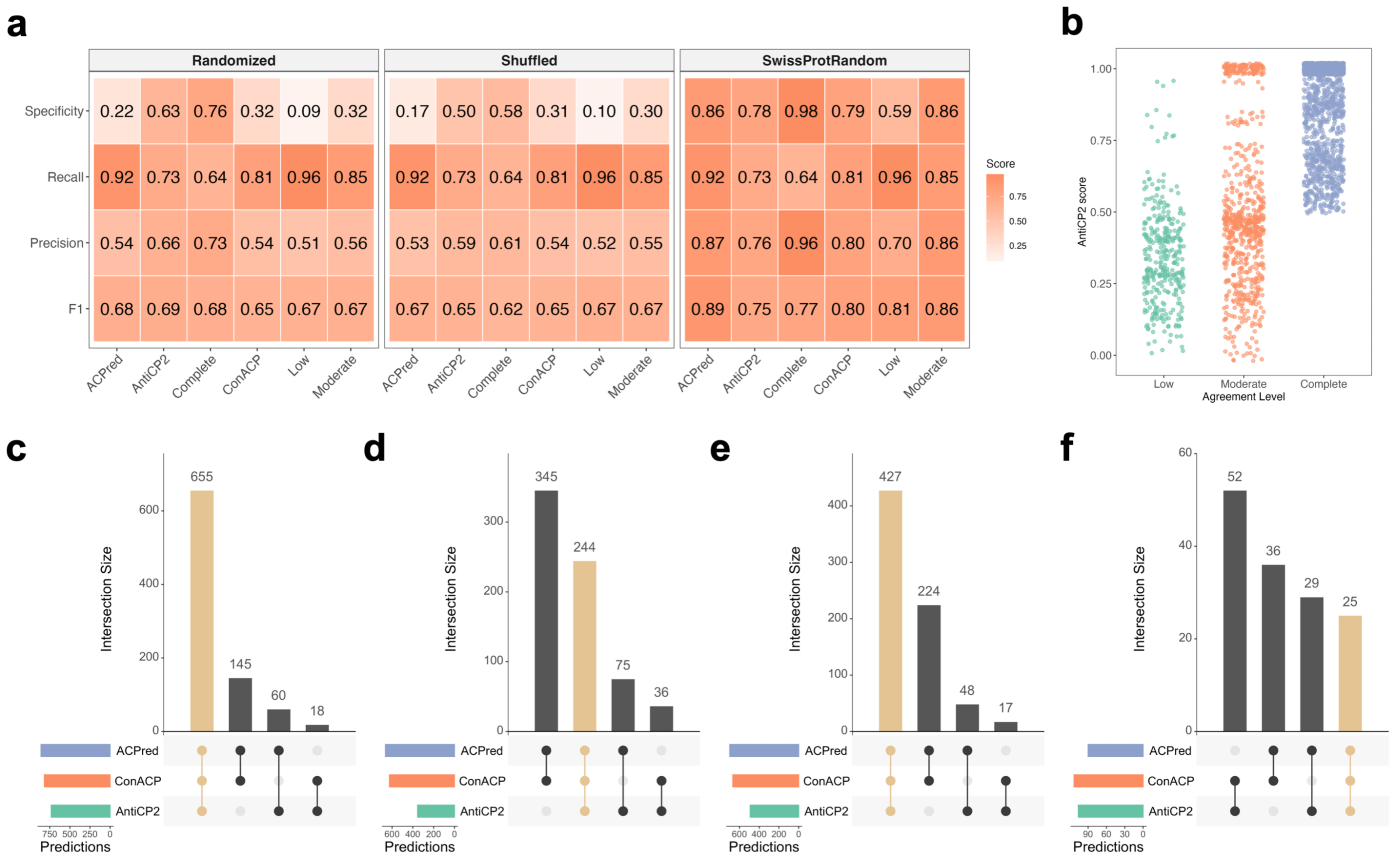
Performance and characteristics of MetaPepticon predictions. (A) Benchmark metrics for individual predictors (AntiCP2, ConACP, ACPred) and consensus thresholds (≥1, ≥2, 3/3) shown as grouped bar plots, displaying F1, precision, recall, and specificity values. (B) AntiCP2 prediction scores *versus* the number of predictors in agreement for validated positive peptides. (C) The overlap of predicted ACPs among AntiCP2, ConACP, and ACPred for positive peptides dataset. (D) The overlap of predicted ACPs among AntiCP2, ConACP, and ACPred for negative peptides dataset. Vertical bars indicate the number of peptides in each intersection, and horizontal bars show the total peptides predicted by each classifier.

Performance metrics varied depending on negative set design, while relative trends across predictors were largely preserved (Fig. 2A). In comparisons against composition-matched randomized negatives, all predictors showed relatively strong apparent performance (Fig. 2A). Among individual models, AntiCP2 exhibited the most balanced profile (precision = 0.66, recall = 0.73, specificity = 0.63; F1 = 0.69), whereas ACPred favored sensitivity (recall = 0.92) at the expense of specificity (0.22). ConACP displayed intermediate behavior with high recall (0.81) but limited specificity (0.32). Under this benchmark, the complete-agreement level of MetaPepticon achieved the highest precision (0.73) but with reduced recall (0.64), illustrating the expected trade-off between confidence and coverage.

When evaluated against shuffled negatives, which control strictly for amino acid composition, performance declined across all predictors (Fig. 2A). AntiCP2 precision decreased from 0.66 to 0.59, and specificity declined from 0.63 to 0.50, while recall remained stable (0.73). Similar patterns were observed for ConACP and ACPred, indicating that a substantial fraction of predictive signal arises from compositional rather than sequence-order–specific features. Under this stringent benchmark, the low-agreement level achieved very high recall (0.96) but negligible specificity (0.10), while the moderate-agreement level showed gains in precision (0.55). On the other hand, the complete-agreement level retained the highest precision (0.61), despite reduced recall (0.64), indicating preferential removal of false positives.

In comparisons against Swiss-Prot-frequency random negatives, all predictors showed strong performance, consistent with pronounced compositional differences between ACPs and typical peptides (Fig. 2A). AntiCP2 achieved a precision of 0.76 and specificity of 0.78, while ACPred combined high recall (0.92) with high specificity (0.86). Under these conditions, the complete-agreement level yielded very high precision (0.96) with reduced recall (0.64), whereas the moderate-agreement level retained high precision (0.86) and specificity (0.86) while substantially improving recall (0.85). In contrast, the low-agreement level maximized recall (0.96) but was accompanied by a marked reduction in specificity (0.59). Together, these results indicate that ensemble agreement amplifies existing compositional separation when negative peptides are compositionally distinct, with moderate- and complete-agreement levels providing a balance between coverage and confidence depending on the desired level of stringency.

Across all negative set designs, the low-agreement level consistently yielded high recall but low precision, indicating substantial false-positive rates. In contrast, moderate-agreement and complete-agreement levels consistently improved precision and specificity with only moderate reductions in recall. Based on benchmarking results, moderate-agreement provides a practical default for balancing sensitivity and precision, while complete-agreement is most appropriate when minimizing false positives is critical.

AntiCP2 scores increased with higher consensus levels, with peptides predicted by multiple algorithms receiving stronger scores (Fig. 2B). Moreover, AntiCP2 scores were positively correlated with the consensus of the other two predictors (Spearman’s *ρ* = 0.45, *p* = 2.2 × 10^−16^), supporting the use of a consensus-based approach. Analysis of prediction overlaps revealed that positive peptides were often identified by multiple classifiers (Fig. 2C), while negative peptides showed little intersection, particularly in composition-matched randomized (Fig. 2D) and Swiss-Prot-frequency random negatives (Fig. 2F).

Together, these results demonstrate that MetaPepticon does not eliminate the intrinsic limitations of current ACP predictors. Instead, it provides a structured framework for controlling prediction performance through agreement thresholds. Across all negative set designs, complete-agreement predictions consistently yielded the highest precision, whereas lower agreement strategies primarily increased sensitivity at the expense of specificity.

### Prediction of candidate anticancer peptides from metagenomic datasets

**Figure 3:**
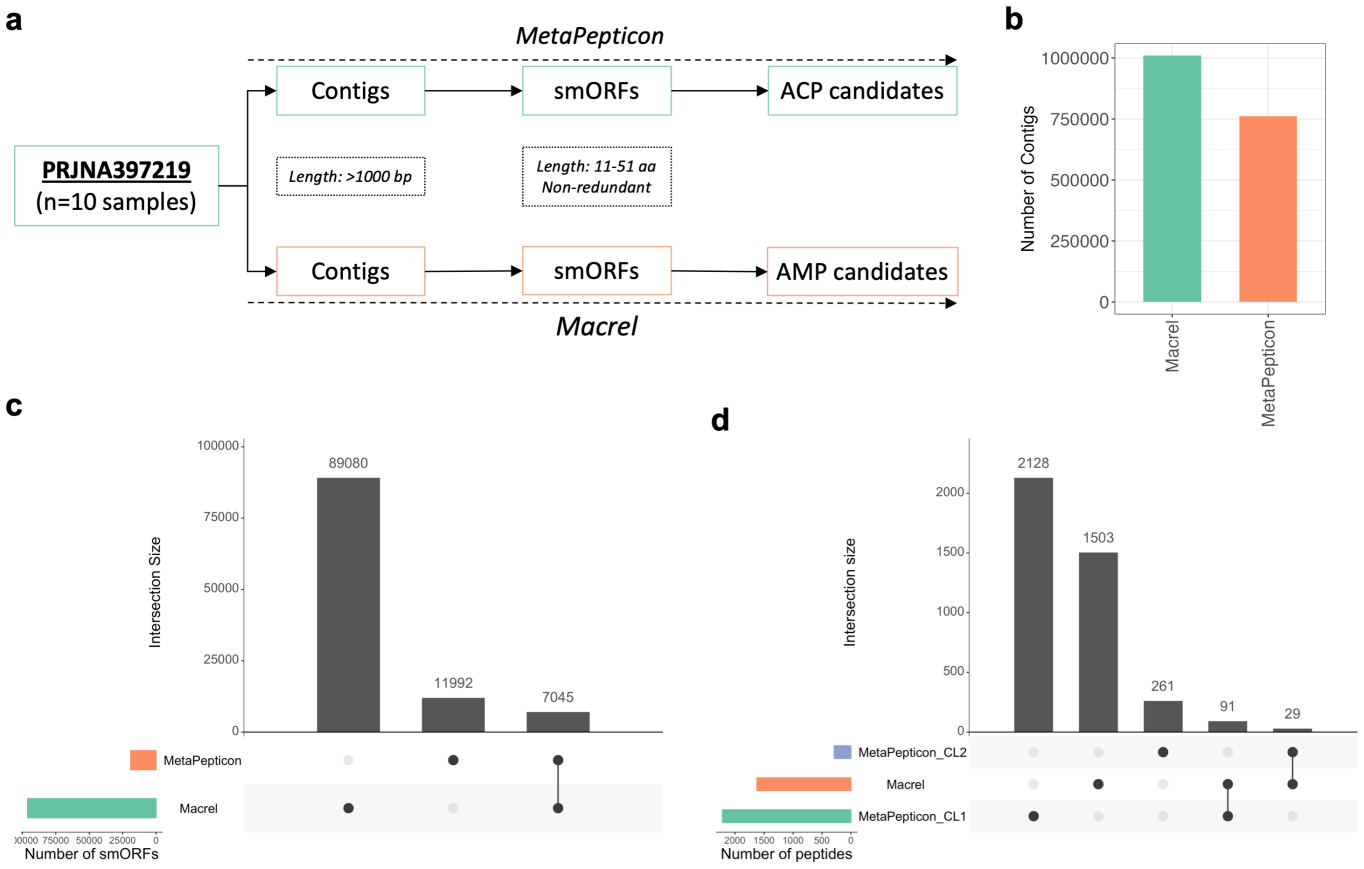
Comparative analysis between MetaPepticon and Macrel. (A) Ten gut metagenome samples were processed with both pipelines, each including de novo assembly, smORF prediction, and functional peptide classification (AMP candidates for Macrel, ACP candidates for MetaPepticon). (B) Total number of contigs generated by each tool. (C) Total and intersecting smORFs predicted by Macrel and MetaPepticon across the dataset. (D) Total number of AMP candidates predicted by Macrel and ACP candidates predicted by MetaPepticon (at ≥1 predictor (CL1) and ≥2 predictor (CL2) agreement levels). Vertical bars indicate the number of smORFs/peptides in each intersection, and horizontal bars show the total number of predictions by each tool.

To illustrate the effect of end-to-end pipeline design on peptide discovery from metagenomic data, we analyzed ten gut metagenome samples from a previously published colorectal cancer cohort (BioProject PRJNA397219, (Hale et al., 2018)) using MetaPepticon. For workflow-level comparison, the same datasets were processed with Macrel (Santos-Júnior et al., 2020), an antimicrobial peptide (AMP) prediction tool that directly accepts raw metagenomic data (Fig. 3A). Because existing ACP predictors require peptide inputs, such a comparison is intended to highlight differences in upstream processing and search-space definition rather than to benchmark ACP prediction accuracy.

MetaPepticon generated 761,673 contigs using metaSPAdes, whereas Macrel produced 1,009,575 contigs (Fig. 3B) using MEGAHIT (Li et al., 2015). At the smORF detection stage, MetaPepticon identified 11,992 unique smORFs, while Macrel predicted 89,080 (Fig. 3B). This order-of-magnitude difference primarily reflects variation in ORF-calling strategies between two pipelines. Both rely on modified versions of Prodigal; however, Macrel employs a more permissive ORF-calling approach primarily based on length filtering, whereas MetaPepticon uses smORFinder, which combines profile hidden Markov models and deep learning models to provide additional filtering layers. Consistent with smORFinder’s design criteria, all 11,992 smORFs retained by MetaPepticon contained annotated ribosome-binding site (RBS) motifs with valid spacer lengths while it is known that Prodigal uses RBS motifs as part of a scoring model rather than a strict filter in its default mode. Within the MetaPepticon smORFs, start codon usage was dominated by ATG (93.3%), with smaller contributions from GTG (5.4%) and TTG (1.3%), consistent with canonical bacterial translation. The median RBS spacer length was 5 bp (mean 5.9 bp), closely matching the optimal Shine–Dalgarno spacing. Although median smORF lengths were comparable between pipelines (∼40 amino acids), the overall length distributions differed significantly (Wilcoxon rank-sum test, *p* < 0.05). Fig. S1 shows a comparative distribution of smORF lengths between MetaPepticon and Macrel outputs.

Among the smORFs detected by MetaPepticon, 7,045 (59%) were also identified by Macrel (Fig. 3C). To assess whether this limited overlap was primarily driven by assembler choice, we re-ran Macrel using the metaSPAdes contigs generated within the MetaPepticon pipeline. Under this configuration, Macrel predicted 177,257 smORFs, of which 11,336 overlapped with MetaPepticon results, corresponding to 95% of MetaPepticon-identified smORFs but only a small fraction of Macrel’s total predictions. These results indicate that assembler choice strongly affects the comparability of smORF sets and confirm that MetaPepticon recovers a conservative subset of high confidence smORFs.

At the functional peptide prediction level, Macrel reported 1,503 AMP candidates, whereas MetaPepticon predicted 2,128 ACP candidates by at least one classifier. When a stricter consensus of at least two classifiers was required, ACP candidates set decreased to 261 (Fig. 3D). Cross-comparison of functional predictions revealed limited overlap, with 91 peptides shared between Macrel AMP candidates and MetaPepticon ACP candidates at the single-predictor level and 29 remaining at the two-predictor consensus level (Fig. 3D). This modest intersection suggests that antimicrobial and anticancer activity predictions, while based on partly overlapping physicochemical and sequence determinants, differ in the relative weighting and combination of these features.

Overall, this analysis demonstrates that differences in assembly, ORF calling, and peptide prioritization strategies substantially shape the peptide search space obtained from metagenomic data. The comparison does not assess ACP prediction accuracy, but instead illustrates how MetaPepticon integrates conservative smORF detection with explicit agreement-based prioritization to enable reproducible identification of ACP candidates directly from raw sequencing datasets.

### Mining public microbial genomes and metagenomes for candidate anticancer peptides

We applied MetaPepticon to explore ACP candidates in large-scale publicly available microbial resources. Specifically, we analyzed 41,171 representative microbial genomes from the proGenomes3 (Fullam et al., 2023) and 4,072,884 peptide sequences from the DBsmORF database (Durrant & Bhatt, 2021). Genomes were processed using the contig module and peptides with the peptide module of MetaPepticon.

**Figure 4:**
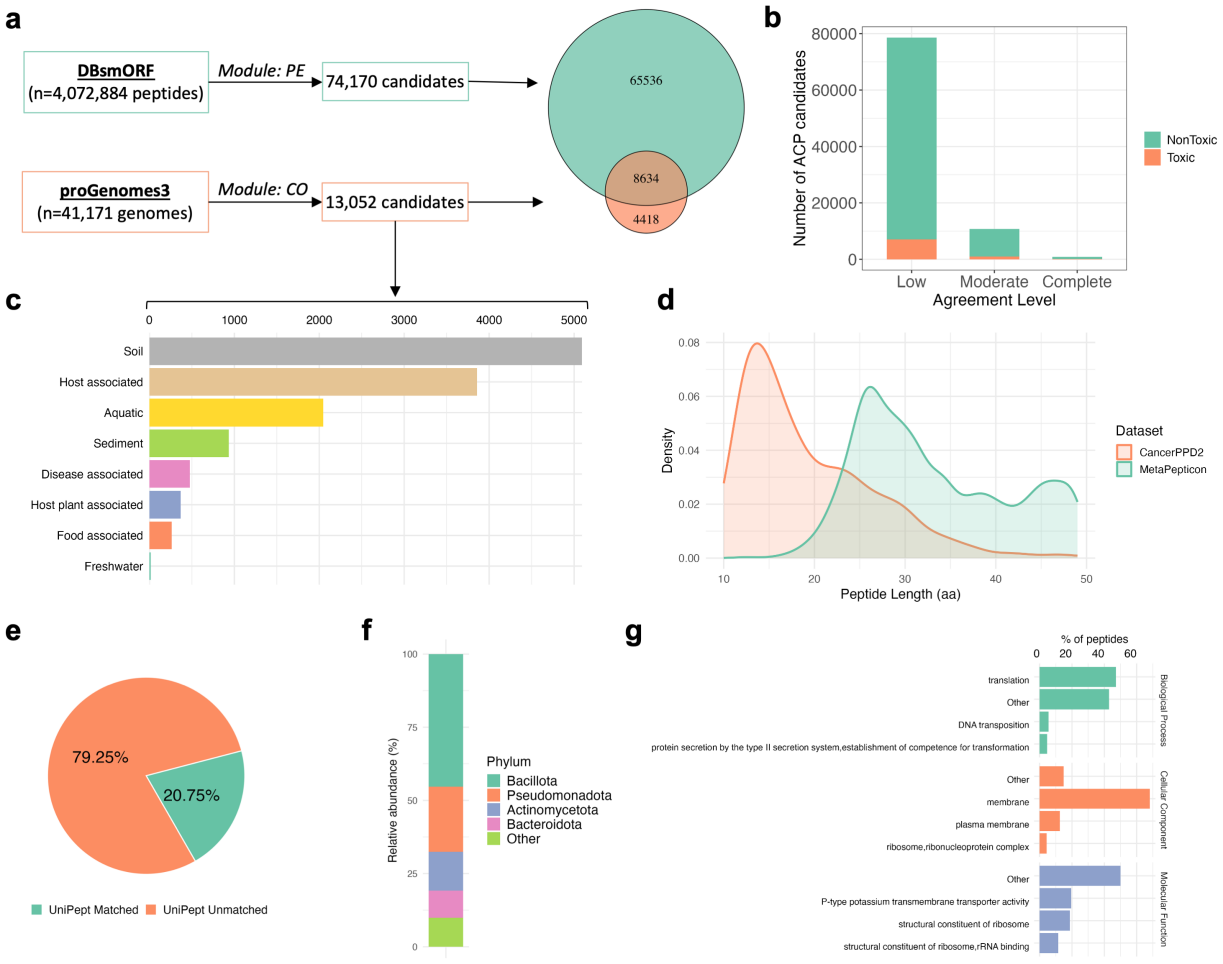
Discovery and characterization of novel ACP candidates using MetaPepticon. (A) Overview of the MetaPepticon pipeline applied to peptides from DBsmORF and contigs from proGenomes3. Venn diagram shows the number of ACP candidates identified from proGenomes3 *versus* DBsmORF, with intersection counts indicating shared ACP candidates. (B) The number of ACP candidates retained at low (≥1 predictor), moderate (≥2 predictors) and complete (3/3 predictors) prediction stringency levels. Colors correspond to toxicity predictions; *y*-axis represents candidate counts on a linear scale. (C) The absolute number of candidates per habitat for proGenomes3-derived peptides. (D) Comparison of ACP candidate lengths predicted by MetaPepticon with peptides from DCTPep and CancerPPD2 databases. (E) Venn diagram depicting the fraction of ACP candidates that could be annotated by UniPept at the ≥1 predictor agreement level. (F) Taxonomic distribution of annotated ACP candidates across bacterial phyla. (G) Gene Ontology (GO) assignments for annotated ACP candidates, shown separately for molecular function, cellular component, and biological process categories.

Across the proGenomes dataset, 13,052 ACP candidates were identified at the low-agreement level, while 74,170 peptides from DBsmORF were predicted as ACP candidates under the same criterion (Fig. 4A). Notably, 8,634 ACP candidates overlapped between the two datasets, consistent with the inclusion of RefSeq-derived genomes and Human Microbiome Project metagenomes in DBsmORF.

After merging results from both databases, we identified 78,588 non-redundant novel ACP candidates. Comparison against existing ACP databases revealed no matches for these peptides. Subsequent toxicity screening indicated that 71,493 candidates were predicted to be non-toxic. Because benchmarking analyses demonstrated that low-agreement predictions exhibit limited precision, candidates were further stratified by predictor agreement. Requiring moderate-agreement yielded 9,822 moderate-agreement non-toxic candidates. Applying the most stringent criterion of complete-agreement resulted in 710 complete-agreement non-toxic ACP canidates (Fig. 4B).

Compared with validated ACPs from CancerPPD2, moderate-agreement ACP candidates exhibited a significantly higher GRAVY index, indicating increased overall hydrophobicity (Wilcoxon rank-sum test, *p* < 0.05; Fig. S2A shows the violin plot comparison of GRAVY index values between the two groups). In contrast, net charge did not differ significantly between the two groups (Wilcoxon rank-sum test, *p* = 0.3; Fig. S2B shows the corresponding net charge comparison). The increased hydrophobicity of MetaPepticon candidates, despite comparable net charge, suggests that these peptides may rely more strongly on membrane-partitioning and insertion mechanisms rather than electrostatic attraction alone, a mode of action previously reported for several membrane-active anticancer peptides (Ghaly et al., 2023).

Habitat-level analysis of ACP candidates predicted from proGenomes revealed that soil microbiomes contribute the largest absolute number of candidates followed by host-associated microbiomes (Fig. 4C). The length distribution of moderate-agreement ACP candidates peaked at 25–30 amino acids, differing from distributions observed in CancerPPD2 database (Fig. 4D). This divergence likely reflects the novelty of microbial peptides, as peptides derived from metagenomes remain underrepresented in current ACP resources, a trend previously noted for AMP candidates identified from global microbiomes (Santos-Júnior et al., 2024).

To assess the novelty of the predicted peptides, moderate-agreement non-toxic ACP candidates were searched against the NCBI non-redundant (nr) protein database using BLASTp. A peptide was considered to have significant homology if at least one hit exhibited ≥50% amino acid identity and ≥90% query coverage. Using these criteria, 5,232 of 9,822 peptides (53.3%) showed detectable similarity to known proteins whereas 4,590 peptides (46.7%) lacked significant similarity.

To gain biological insight, we performed taxonomic and functional annotation of the moderate-agreement ACP candidates using UniPept (Vande Moortele et al., 2025). Only 21% of peptides could be taxonomically annotated (Fig. 4E), revealing diverse microbial origins dominated by *Bacillota* (45.3%), *Pseudomonadota* (22.3%), *Actinomycetota* (13.3%), *Bacteroidota* (9.3%) (Fig. 4F). Several taxa enriched among the predicted peptides, particularly *Bacillota* and *Pseudomonadota*, are known producers of ribosomally synthesized short cationic peptides with amphipathic structures, features shared with many experimentally validated ACPs (Santos-Júnior et al., 2024; Zhang et al., 2025). Prior studies have demonstrated that such peptides can exert anticancer effects through membrane destabilization, mitochondrial perturbation, and the induction of apoptosis (Hoskin & Ramamoorthy, 2008; Wang et al., 2022).

Functional profiling indicated broad molecular diversity, with ribosomal, translational, and membrane-associated peptides among the most enriched categories (Fig. 4G). This enrichment is consistent with two commonly described ACP interaction modes. First, peptides associated with ribosomal or translation-related functions may interfere with protein synthesis or cellular stress-response pathways, which are particularly critical in rapidly proliferating cancer cells (Schweizer, 2009; Tornesello et al., 2020). Second, membrane-associated annotations align with the preferential interaction of many ACPs with anionic cancer cell membranes, a property attributed to altered lipid composition and surface charge in malignant cells (Schweizer, 2009; Borrelli et al., 2018). These taxonomic and functional patterns support the biological plausibility of the predicted ACP candidates without implying direct experimental validation.

## Discussion

MetaPepticon is a modular bioinformatics pipeline for identifying anticancer peptide (ACP) candidates from raw sequencing data, contigs, or peptide sequences. It integrates genome assembly, open reading frame prediction, ACP prediction and toxicity assessment into a single automated workflow, addressing key bottlenecks in scalable ACP discovery. By combining multiple prediction algorithms with toxicity profiling, MetaPepticon enables flexible yet high-confidence candidate selection and supports prioritization for downstream experimental validation.

A key strength of MetaPepticon lies in its ability to bridge microbiome datasets with functional peptide discovery. The pipeline enables systematic mining of large microbial datasets, uncovering substantial numbers of novel ACP candidates across diverse microbial sources. Application to extensive public datasets demonstrates its capacity to identify previously uncharacterized peptides, expanding the repertoire of potential therapeutic candidates.

Despite its usefulness and effectiveness in ACP prediction, MetaPepticon still has certain limitations. MetaPepticon depends on existing ACP predictors and therefore inherits their biases and accuracy constraints. Strict agreement thresholds may reduce sensitivity by excluding some true positives and experimental validation of predicted candidates was beyond the scope of this work. In addition, sequence-based approaches may not be suitable for non-canonical ACPs.

Future extensions of MetaPepticon may include the incorporation of emerging machine learning–based predictors, complementary structure-based or biophysical analyses, and improved support for non-bacterial peptides. Additional developments could focus on enhancing accessibility through containerization or graphical interfaces, with the broader aim of facilitating adoption while maintaining methodological transparency.

## Conclusions

MetaPepticon is a modular bioinformatics pipeline designed for the discovery of ACP candidates from raw sequencing datasets, as well as contigs and peptide sequences. It streamlines the identification process by integrating genome assembly, open reading frame prediction, peptide annotation, and toxicity assessment within a single automated workflow. By incorporating multiple prediction algorithms, MetaPepticon allows users to balance broad discovery with high-confidence candidate selection, while toxicity predictions facilitate prioritization for experimental validation.

## Supplemental Information

10.7717/peerj.20990/supp-1 Supplemental Information 1 Length distribution of small open reading frames predicted by Macrel and MetaPepticon The frequency of predicted smORFs across peptide length ranges for each method. Differences in the distributions highlight variations in prediction behavior between Macrel and MetaPepticon, particularly with respect to preferred peptide length ranges and the representation of shorter versus longer smORFs.

10.7717/peerj.20990/supp-2 Supplemental Information 2 Comparison of physicochemical properties between validated anticancer peptides (ACPs) from CancerPPD2 and moderate-agreement ACP candidates (A) Distribution of GRAVY index values, showing significantly higher hydrophobicity in moderate-agreement ACP candidates compared with validated ACPs (Wilcoxon rank-sum test, *p* < 0.05). (B) Distribution of net charge values, indicating no significant difference between the two groups (Wilcoxon rank-sum test, *p* = 0.3).

## Additional Information and Declarations

### Data Availability

The following information was supplied regarding data availability: MetaPepticon is available at: https://github.com/arikanlab/MetaPepticon
The public microbial genomes and peptide sequences analyzed in this study are available at: https://progenomes.embl.de/download.cgi
The RefSeq and HMP sequences are available at DBsmORF: http://104.154.134.205:3838/DBsmORF/
- All sequences in both RefSeq and HMP databases were utilized. The gut metagenome samples analyzed are available at NCBI: PRJNA397219.
