# Supplementary figures and images for: MetaPepticon: automated prediction of anticancer peptides from microbial genomes and metagenomes
Source: PeerJ. 2026 Mar 27;14:e20990. doi: 10.7717/peerj.20990 (PMC13034871; doi:10.7717/peerj.20990)

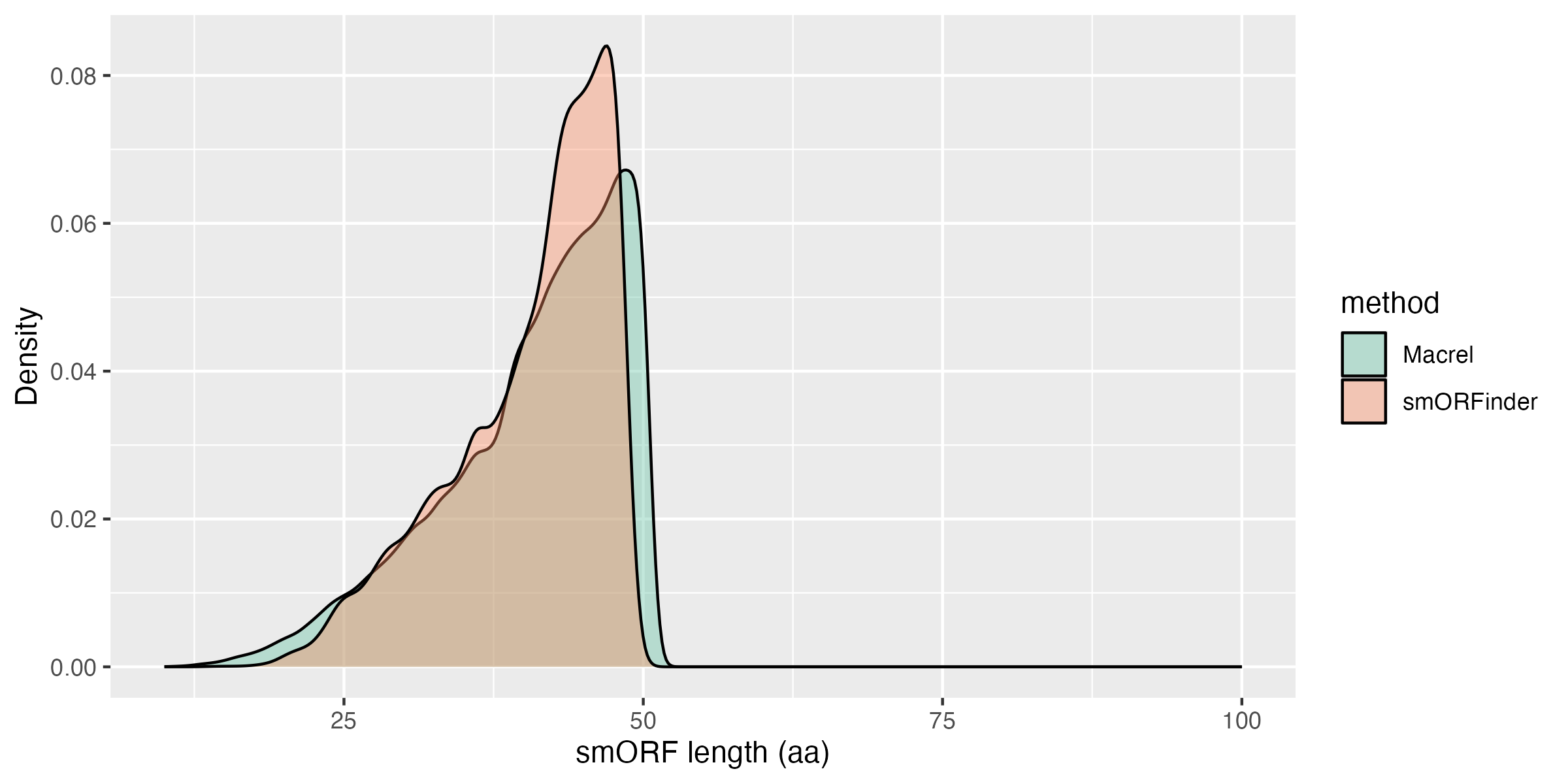

Supplement: Supplemental Information 1 — The frequency of predicted smORFs across peptide length ranges for each method. Differences in the distributions highlight variations in prediction behavior between Macrel and MetaPepticon, particularly with respect to preferred peptide length ranges and the representation of shorter versus longer smORFs. [file peerj-14-20990-s001.png]

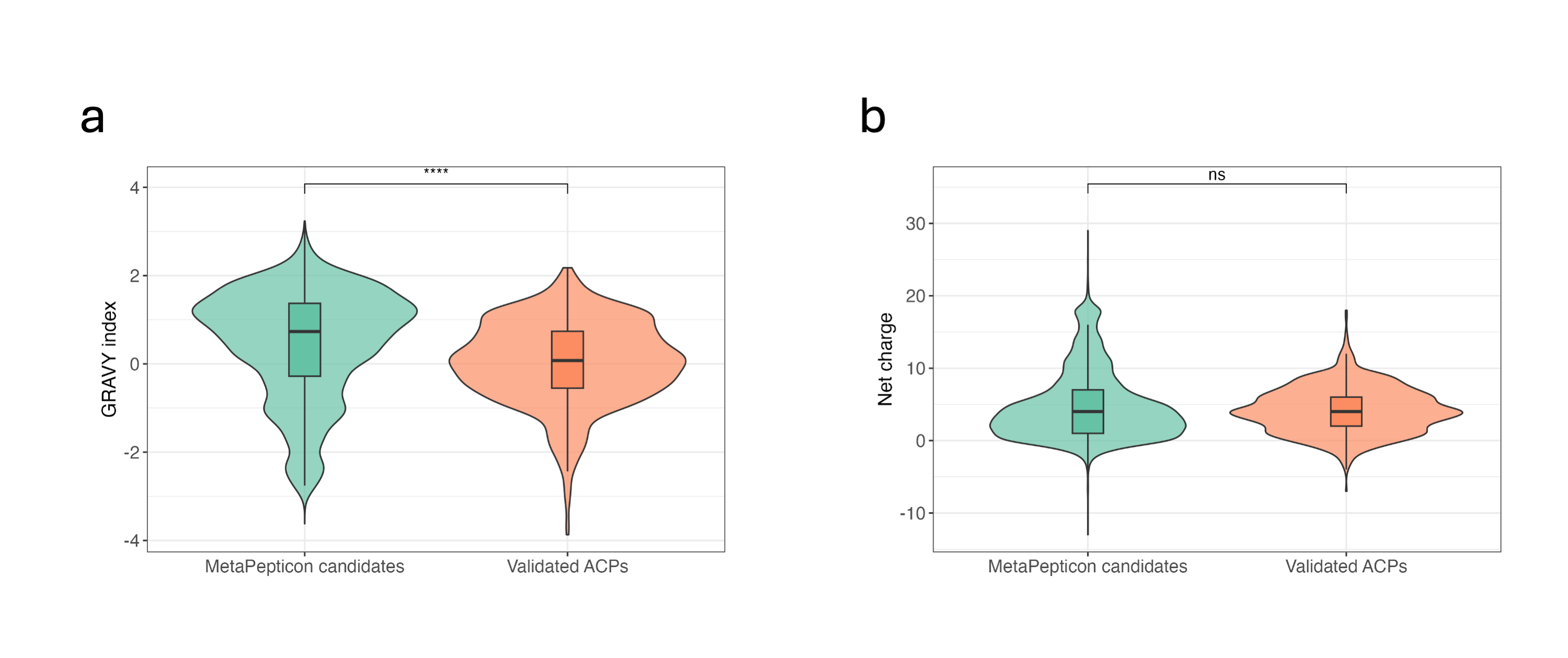

Supplement: Supplemental Information 2 — (A) Distribution of GRAVY index values, showing significantly higher hydrophobicity in moderate-agreement ACP candidates compared with validated ACPs (Wilcoxon rank-sum test, p < 0.05). (B) Distribution of net charge values, indicating no significant difference between the two groups (Wilcoxon rank-sum test, p = 0.3). [file peerj-14-20990-s002.png]
